# Topology optimization design of the height-adjustment mechanism of a shearer cable bending test machine

**DOI:** 10.1371/journal.pone.0354513

**Published:** 2026-08-25

**Authors:** Beilin Dong, Shutian Gong, Yuzheng Zhu, Lijuan Zhao, Xinjiang Yu, Feida Wang, Yuxiang Wang, Dongyang Wang, Tiangu Wu, Zhanpeng Zhang, Zhongjian Bai, Shuai Zhang

**Affiliations:** 1 Changlong Cable Manufacturing Co., Ltd., Shandong Yankuang Group, Jining, Shandong, China; 2 School of Mechanical Engineering, Liaoning Technical University, Fuxin, Liaoning, China; 3 Liaoning Professional Technology Innovation Center of Hydraulic Transmission and Control, Fuxin, Liaoning, China; TU Dublin Blanchardstown Campus: Technological University Dublin - Blanchardstown Campus, IRELAND

## Abstract

The bending-resistance test of shearer cables should be consistent with actual underground operating conditions, in which the cable dragging height, bending radius and contact state vary with different shearer models and cable specifications. Existing bending test machines generally perform dragging tests at a fixed height, which limits their ability to reproduce the real service state of shearer cables. To address this problem, a hydraulically assisted height-adjustment system based on rack-and-pinion transmission was designed in this study. Dynamic simulation, fatigue-life prediction and lightweight design of the height-adjustment mechanism were then carried out. A three-dimensional model of the height-adjustment mechanism was established according to the matching principle of shearer cable dragging height. An MCPT-1.9/3.3 cable with a specification of 3 × 185 + 1 × 95 + 4 × 10 was selected as the research object. A layered equivalent modeling method was adopted, and tensile tests of the strands and control-core conductors were conducted to determine the material parameters. The equivalent elastic modulus and Poisson’s ratio of the assembled cable were obtained as 28,503 MPa and 0.394, respectively. A rigid–flexible coupled dynamic model of the height-adjustment mechanism was established in RecurDyn to analyze the dynamic stresses of the gear, rack, guide column, limit block and slider. Fatigue-life prediction was performed for the guide-column limiting-hole region under alternating loads. Topology optimization was further conducted using the variable-density method in OptiStruct. The results show that the maximum equivalent stresses of the gear and rack during the height-adjustment process are 73.27 MPa and 42.82 MPa, respectively. During the dragging process at a height of 1.8 m, the maximum equivalent stresses of the guide column, limit block and slider are 65.11 MPa, 45.89 MPa and 17.73 MPa, respectively, which are all lower than the allowable stress of the material. Based on the rainflow counting method and the Palmgren–Miner criterion, the fatigue life of the guide column is calculated. After topology optimization, the masses of the guide column, limit block, supporting plate, slider and guide-column sleeve are reduced by 37.7%, 55.6%, 36.4%, 51.9% and 5.6%, respectively. The total mass of the height-adjustment mechanism is reduced by 132.6 kg. The maximum equivalent stresses of the guide column, limit block and slider are reduced by 23.3%, 26.4% and 34.5%, respectively, and the optimized guide column still satisfies the fatigue-life design requirement. This study provides a theoretical basis for the optimized design of shearer cable bending test machines.

## 1. Introduction

Shearer cables perform the dual functions of power transmission and control-signal transmission for coal shearers. Under underground dragging conditions, they are subjected to long-term combined effects such as reciprocating bending, tensile loading and contact compression, which can readily lead to conductor fracture, insulation wear and fatigue failure [1–3]. Accurate evaluation of the bending resistance of shearer cables is therefore essential not only for ensuring the delivery quality of cable products, but also for maintaining the continuity and safety of coal-mine production.

Existing shearer cable bending test machines mostly adopt a fixed dragging height and can only conduct bending tests under a single geometric boundary condition. As a result, they cannot realistically reproduce the dragging height, bending radius and contact state corresponding to different specifications of shearer cables under underground operating conditions. With the development of shearers toward intelligent and large-scale equipment, the cross-sectional area and number of cores of supporting cables continue to increase. Test equipment must therefore have stronger adaptability to operating conditions; otherwise, deviations between test results and actual service life will persist.

A number of studies have been carried out on cable bending test equipment and topology optimization based on the OptiStruct variable-density method. Feng et al. [4] developed an early cable bending-life test machine. Ma et al. [5] developed a silent and wear-resistant rail device to address the problems of high noise, short service life and the need for complete replacement after rail damage in conventional repeated-bending test machines. Jin et al. [6] designed a cable bending test device according to the MT818–2009 standard and performed modal analysis of the first ten modes of the frame using ABAQUS. Jacob Steendam et al. [7] developed an alternating bending test device for Labco GmbH in Germany, which supports multi-specimen testing, continuous power-off monitoring and operation over a wide temperature range. Erena Guardia D. et al. [8] designed a cable fatigue test device capable of applying combined axial loads and bending moments. Ma [9] investigated the calibration method of a cable conduit bending test machine, established a calibration measurement model system and analyzed the sources of uncertainty. Zhu et al. [10] and Qian et al. [11] proposed improved schemes for cable bending-point fixation, adaptation to cables with different diameters and torsional bending performance testing. In terms of topology optimization, McClanahan et al. [12] optimized the internal structure of compressor blades using OptiStruct, reducing the mass of hollow blades by 59.8% and significantly decreasing stress. Serrano et al. [13] conducted topology optimization and additive manufacturing of a camera bracket for a 3U CubeSat, reducing its mass from 1.15 kg to less than 150 g. Sahu et al. [14] optimized the structure of a space reflector while maintaining its optical performance. Lee Kibum et al. [15] performed topology optimization of the upper support of an automotive radiator using plastic materials, achieving simultaneous improvements in structural strength and lightweight performance. Xu et al. [16], based on topology optimization theory, implemented lightweight design for the wheel hub of a certain vehicle model, formulated various lightweight schemes under different constraint conditions, and conducted comparative analysis. Shi et al. [17] took specific permanent magnet synchronous motor housings as research objects, using OptiStruct software for finite element analysis and verifying the physical housing performance through modal testing. Gao et al. [18] used the variable density topology optimization method in OptiStruct software to analyze the structure of MCU brackets, obtaining the optimal material distribution scheme while ensuring modal performance is not lower than the original model. After optimization, bracket weight is reduced by 13.5%, and unit cost is reduced by about 6.36 yuan, achieving lightweighting, cost reduction, and efficiency improvement. Bian et al. [19] used a combination of topological and dimensional optimization methods to carry out lightweight design of the turnover aluminum box, constructing a multi-condition topology optimization mathematical model based on eclectic programming. Through eclectic planning topology optimization, a new box structure was formed, achieving a 10.15% reduction in box mass under the same wall thickness conditions. Hao et al. [20] took a rail vehicle antenna bracket as the research object, utilizing the OptiStruct software platform to collaborate on topology and dimensional optimization design. The optimized new antenna bracket structure achieves a significant 68.1% reduction in mass compared to the original structure. Chen et al. [21] aimed to solve the problem of loose cylinder mounting bolts during the operation of feed briquetting machine prototypes and to improve the lightweight level of the frame. Using the finite element software Hypermesh to establish finite element models, defining design space, applying loads and constraints, and simulating force conditions, topology optimization was performed using OptiStruct. After optimization, the model’s maximum concentrated stress was reduced, achieving a lightweight effect of 33.8%.

Based on an enterprise-commissioned project, this study investigates a shearer cable bending test machine with height-adjustment capability. The structure of the height-adjustment system is designed, equivalent cable and rigid–flexible coupled dynamic models of the height-adjustment mechanism are established, the stresses and strains of key components are analyzed, the fatigue life of the guide column is predicted, and topology optimization is conducted.

## 2. Model construction and parameter settings of the height-adjustment mechanism of the shearer cable bending test machine

The height-adjustment mechanism of the shearer cable bending test machine designed by the project team is driven by a permanent-magnet synchronous motor. Through rack-and-pinion transmission, the guide column is driven to move vertically, thereby achieving a height-adjustment range of 0.4–1.8 m.

**Fig 1:**
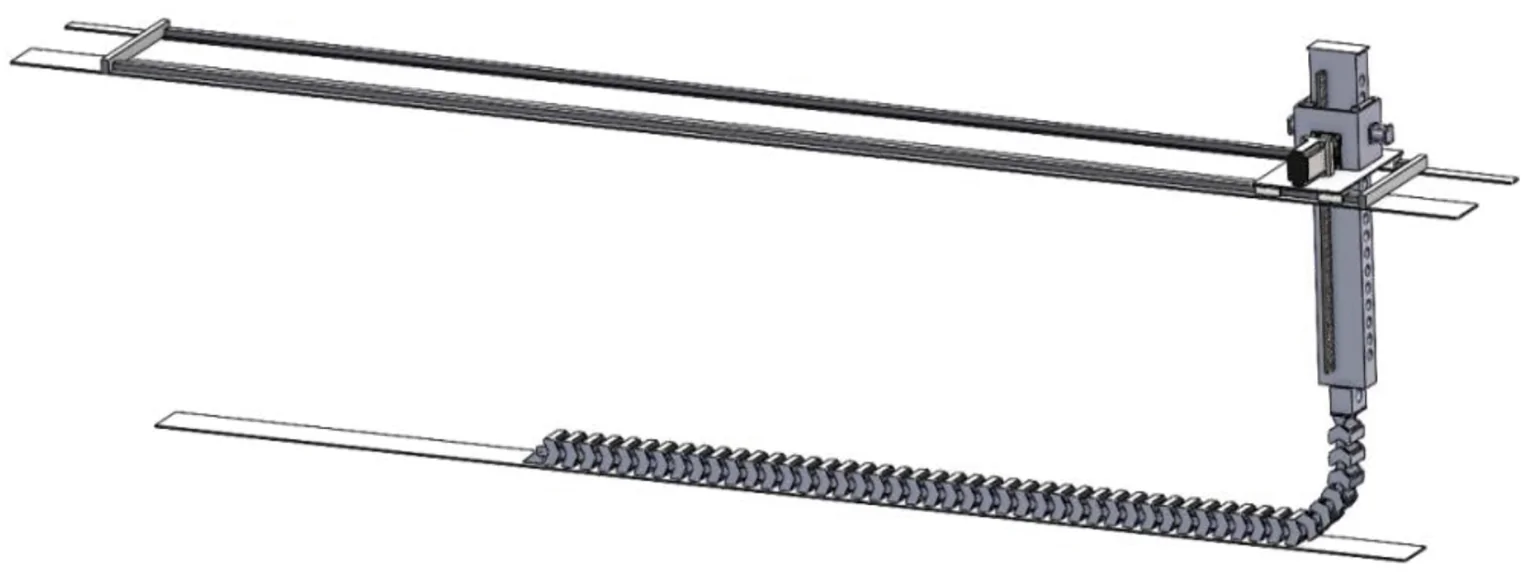
Model of the height-adjustment mechanism of the shearer cable bending test machine.

Based on SolidWorks, three-dimensional solid models of the height-adjustment mechanism of the shearer cable bending test machine and the dragged cable were established, as shown in Fig 1. The models were imported into the dynamic simulation software RecurDyn in STEP format for material assignment, constraint definition, driving-function setting and contact-condition definition.

**Fig 2:**
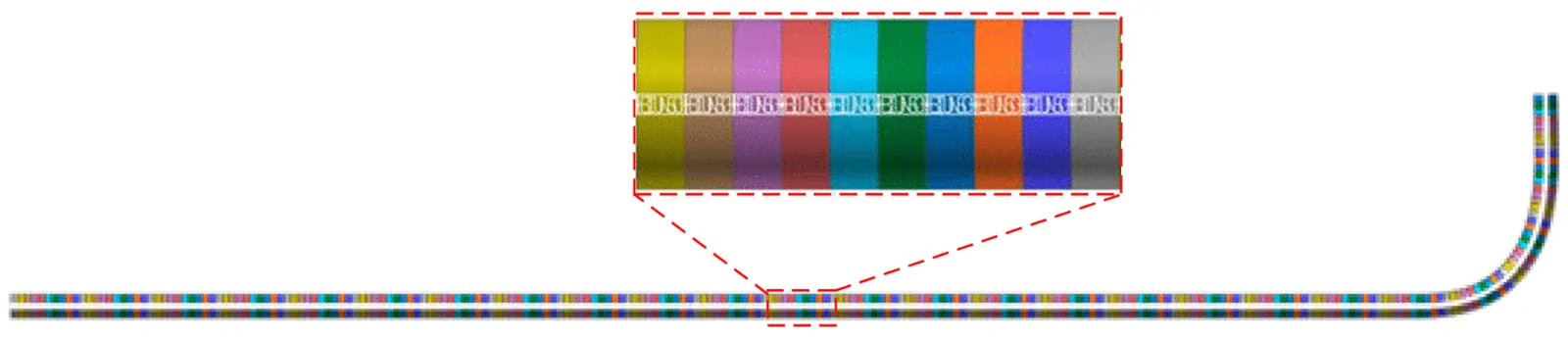
Equivalent cable model.

Using an equivalent cable method to reproduce the actual operating conditions of shearer cables can effectively solve the problems caused by their complex structure, high difficulty in overall finite-element modeling and long simulation time. An MCPT-1.9/3.3 cable with a specification of 3 × 185 + 1 × 95 + 4 × 10 was selected as the engineering object. Quasi-static tensile tests were conducted on the strands and control-core conductors to determine their elastic moduli [22,23], and equivalent treatment was then performed [24]. The equivalent elastic modulus and Poisson’s ratio of the assembled cable were obtained as 28,503 MPa and 0.394, respectively. A three-dimensional model of the equivalent cable was established and imported into RecurDyn, as shown in Fig 2.

**Fig 3:**
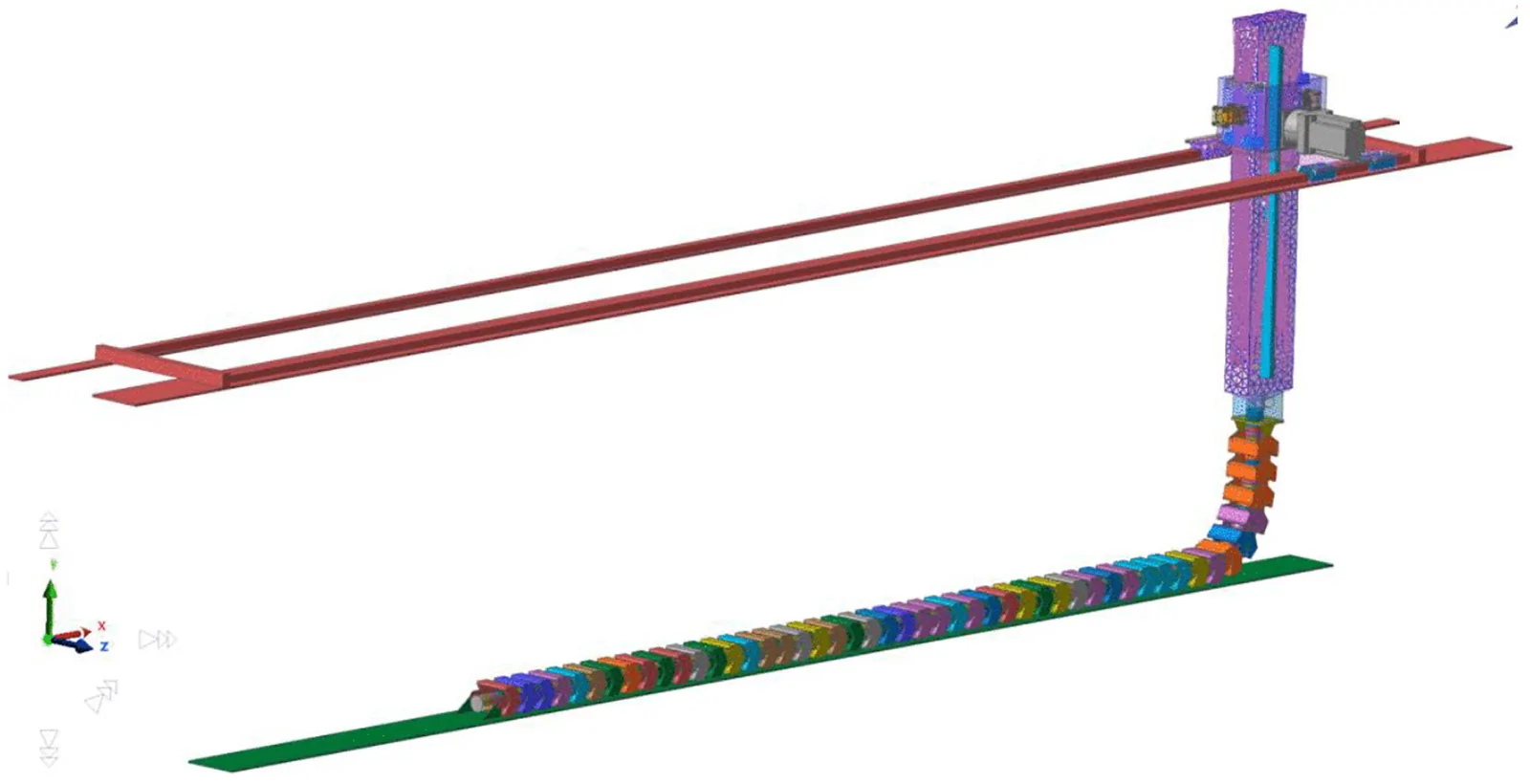
Overall rigid–flexible coupled model of the height-adjustment mechanism of the shearer cable bending test machine.

The G-Manager module in RecurDyn was used to perform F-Flex flexible-body modeling of the height-adjustment mechanism of the shearer cable bending test machine based on the absolute nodal coordinate formulation. To improve simulation efficiency, the R-Flex method based on modal reduction technology in RecurDyn was adopted [25]. The R-Flex flexible bodies were converted using the Dynamis solver. The resulting rigid–flexible coupled model of the height-adjustment mechanism of the shearer cable bending test machine is shown in Fig 3.

## 3. Dynamic simulation analysis of the height-adjustment mechanism of the cable bending test machine

The height-adjustment state and the cable dragging-bending state were selected as two simulation conditions. Dynamic simulations were carried out using RecurDyn.

### 3.1 Analysis of simulation results during height-adjustment motion

**Fig 4:**
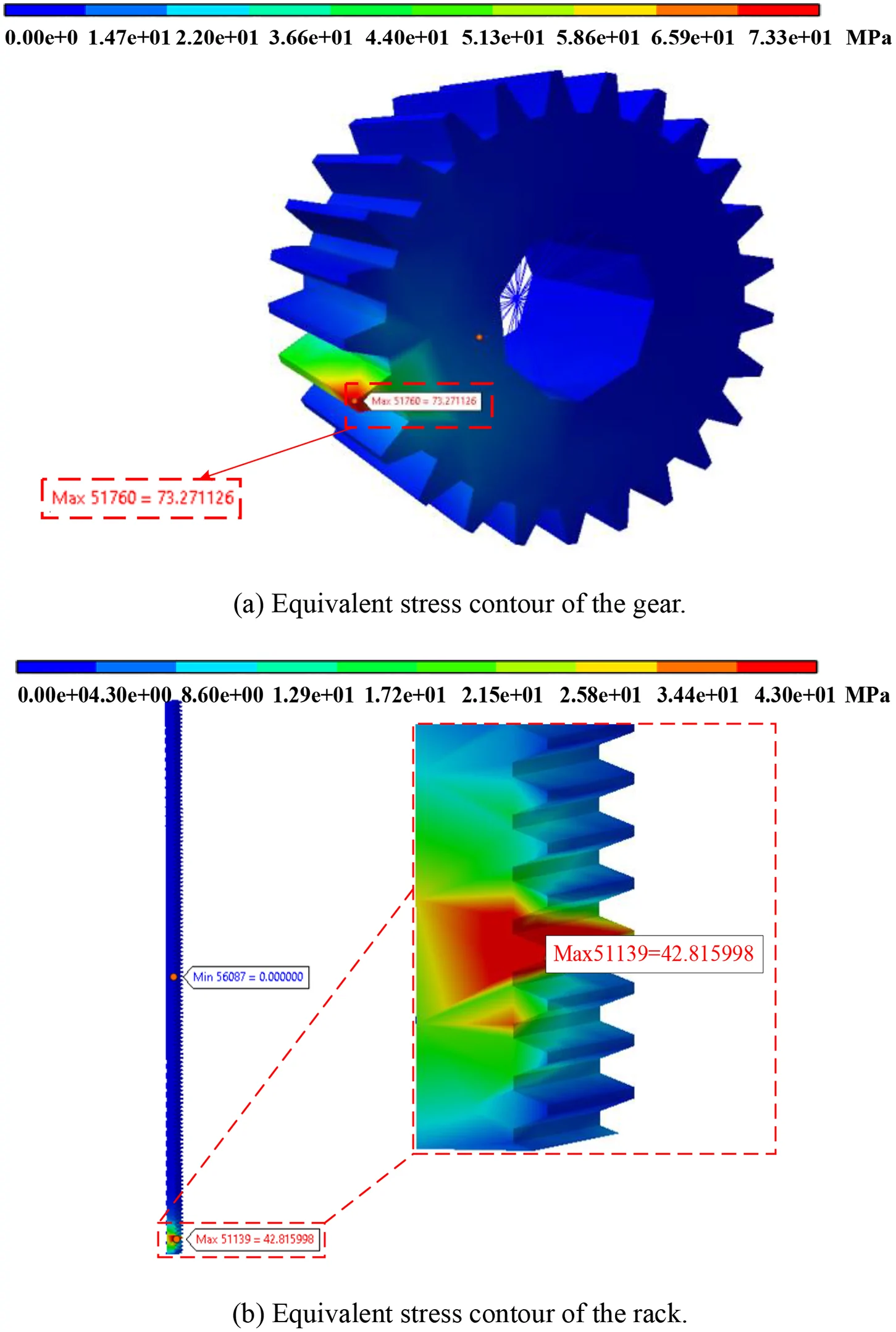
Equivalent stress contours of the gear and rack during height-adjustment motion. (a) Equivalent stress contour of the gear. (b) Equivalent stress contour of the rack.

A driving function was applied to make the gear rotate at an angular velocity of 0.25 rad/s. The gear drove the rack and guide column to move axially, thereby raising the dragging end from 0.7 m to 1.8 m. After the simulation was completed, the equivalent stress contours of the gear and rack were obtained, as shown in Fig 4.

As shown in Fig 4, the maximum equivalent stresses of the gear and rack were 73.27 MPa and 42.82 MPa, respectively. Both values were lower than the allowable material stress of 142 MPa, indicating that the strength requirement was satisfied.

### 3.2 Analysis of simulation results during dragging motion

**Fig 5:**
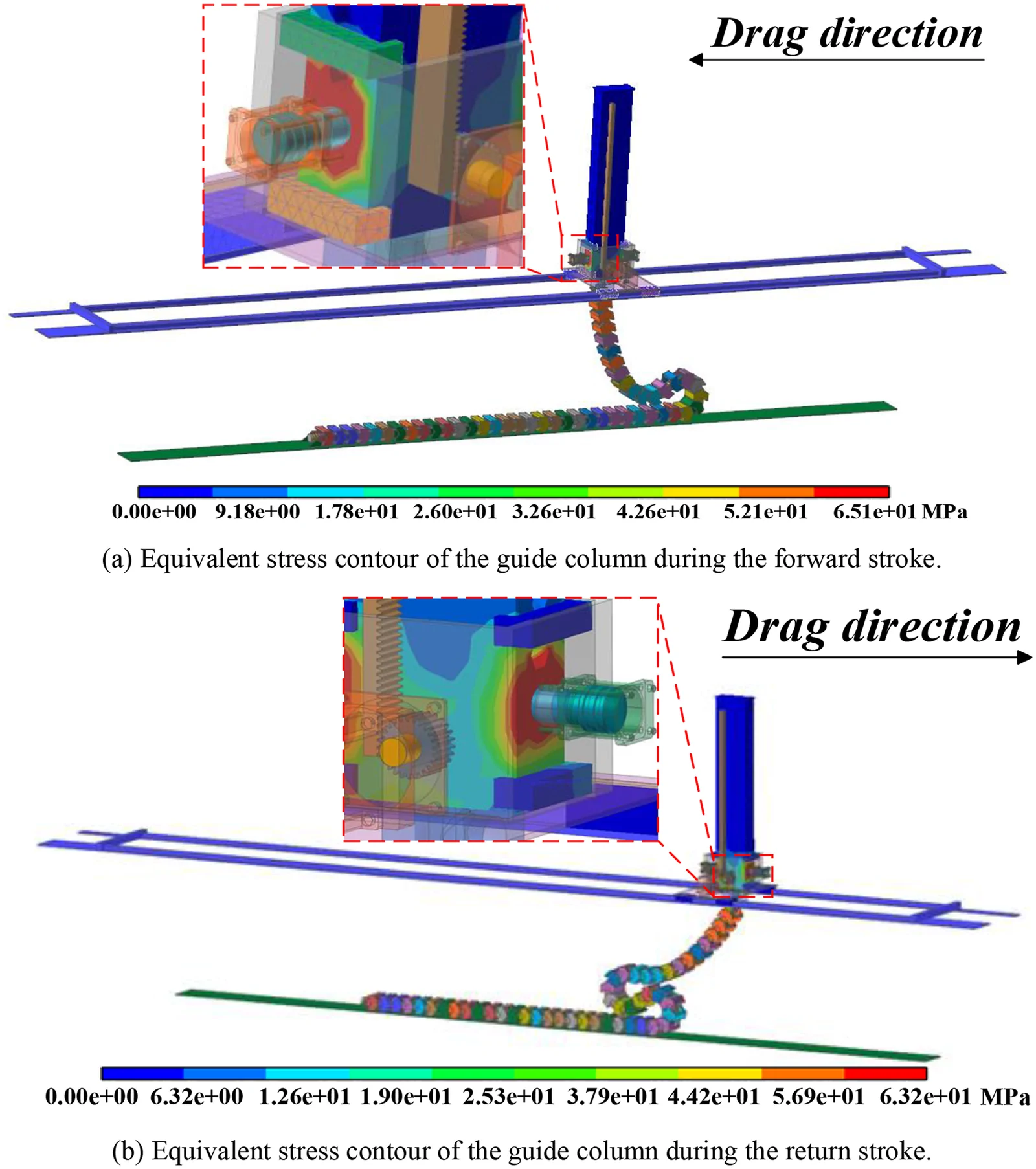
Equivalent stress contours of the guide column. (a) Equivalent stress contour of the guide column during the forward stroke. (b) Equivalent stress contour of the guide column during the return stroke.

**Fig 6:**
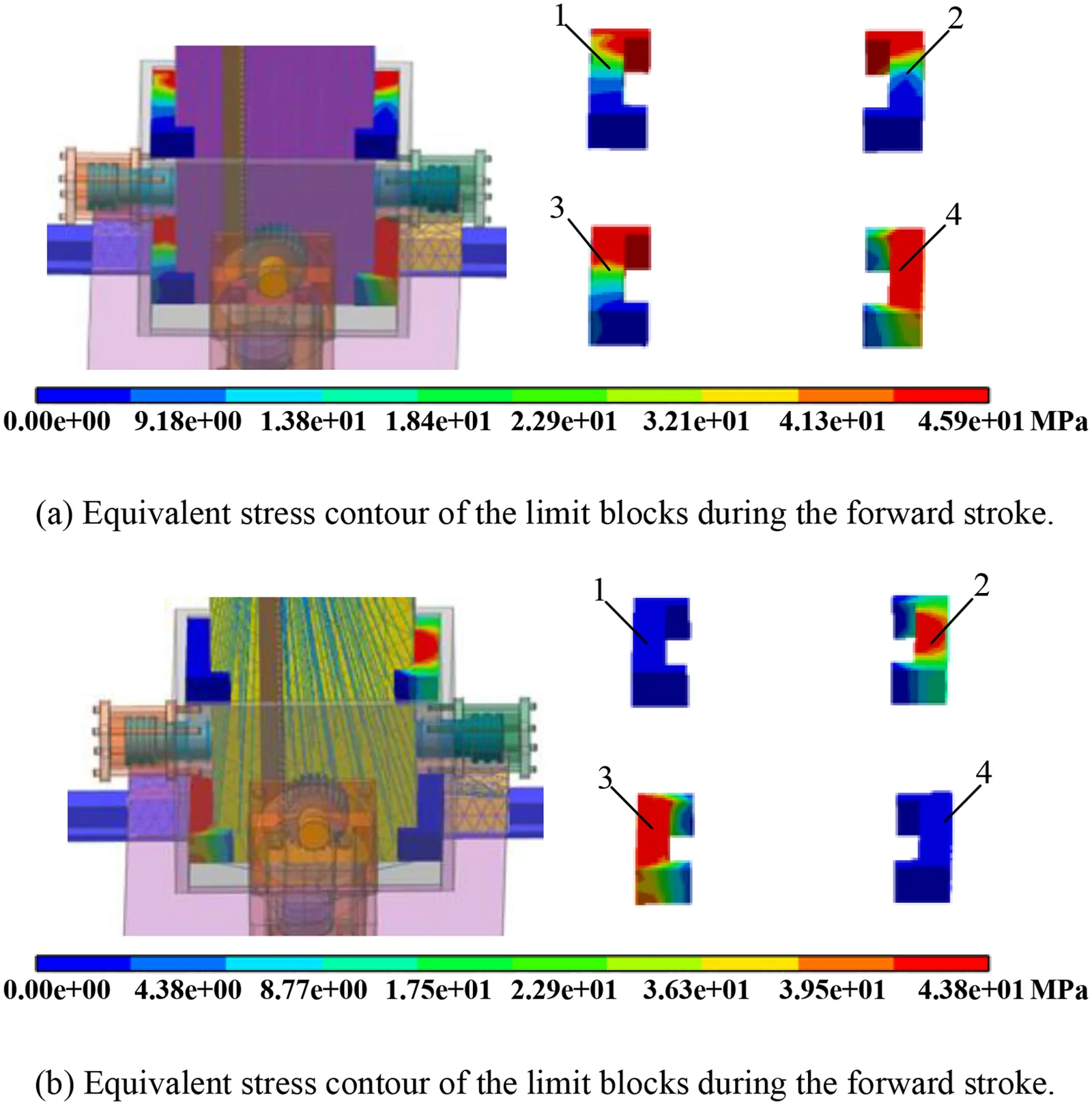
Equivalent stress contours of the limit blocks. (a) Equivalent stress contour of the limit blocks during the forward stroke. (b) Equivalent stress contour of the limit blocks during the forward stroke.

**Fig 7:**
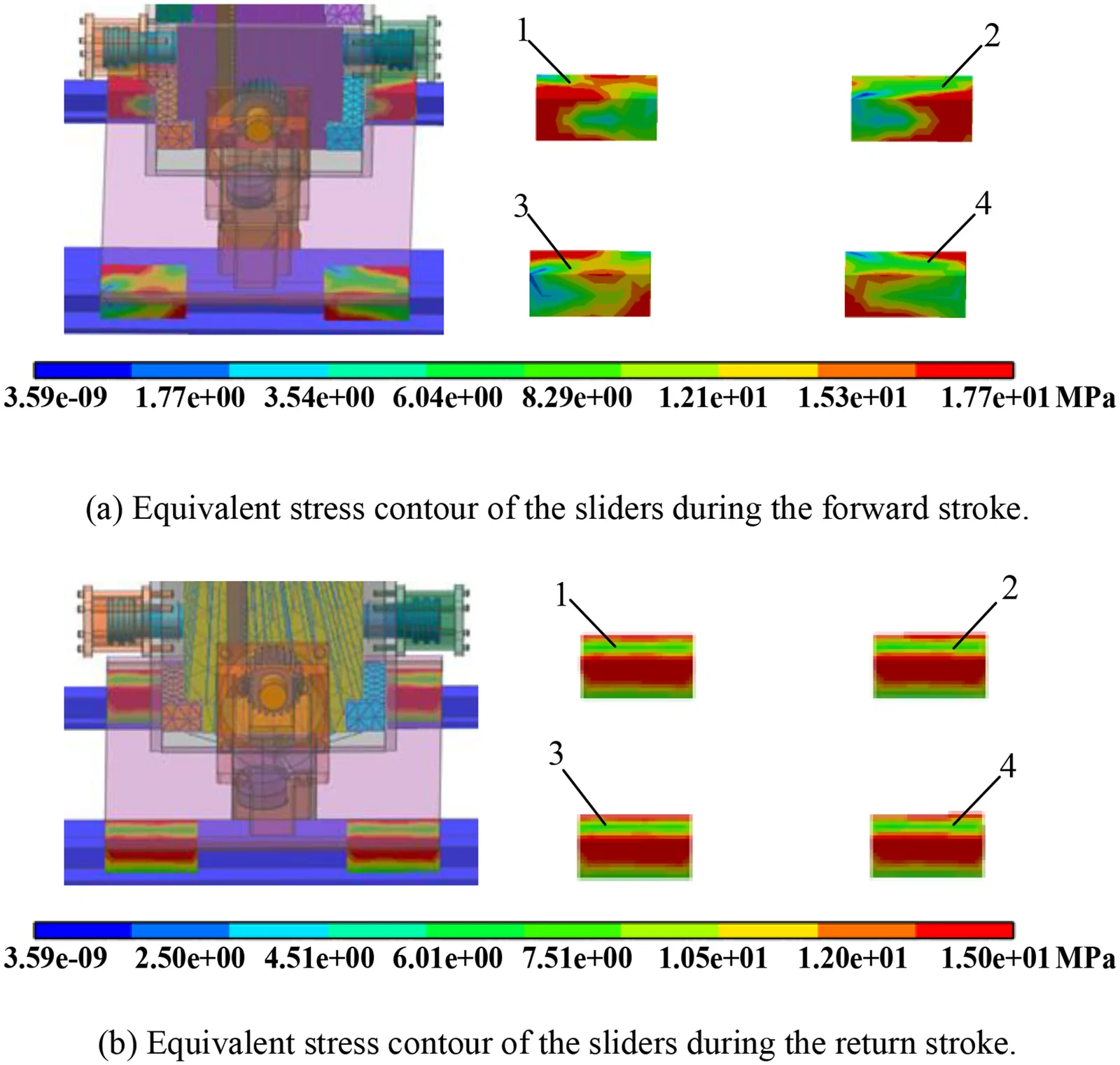
Equivalent stress contours of the sliders. (a) Equivalent stress contour of the sliders during the forward stroke. (b) Equivalent stress contour of the sliders during the return stroke.

A driving function was applied to simulate the cable dragging–bending state. The equivalent stress contours of the guide column, limit blocks and sliders during the forward and return strokes were obtained, as shown in Figs 5–7.

According to the simulation results, the maximum equivalent stresses of the guide column during the forward and return strokes were 65.11 MPa and 63.24 MPa, respectively. The four limit blocks were subjected to different forces during the forward and return simulations. During the forward stroke, the maximum equivalent stress nodes appeared on limit block 1 and limit block 4 for most of the simulation time. The maximum equivalent stress during the entire forward-stroke simulation occurred on limit block 4, with a value of 45.89 MPa. During the return stroke, the equivalent stresses of limit blocks 2 and 3 were significantly higher than those of limit blocks 1 and 4. The maximum equivalent stress during the entire return-stroke simulation occurred on limit block 3, with a value of 43.77 MPa. The maximum stress values of the four sliders were basically similar during the forward and return simulations. During the forward stroke, slider 1 produced the maximum equivalent stress of 17.73 MPa. During the return stroke, slider 2 produced the maximum equivalent stress of 15.04 MPa.

The above dynamic simulation results show that, compared with the other components, the guide column was subjected to the highest maximum equivalent stress during the forward stroke. In addition, alternating cyclic loads acted between the guide column and the hydraulic rod during operation, whereas the equivalent-stress curves of the other components were relatively smooth. Therefore, fatigue-life prediction was carried out for the guide column during the forward stroke in this study.

### 3.3 Fatigue-life simulation analysis

**Fig 8:**
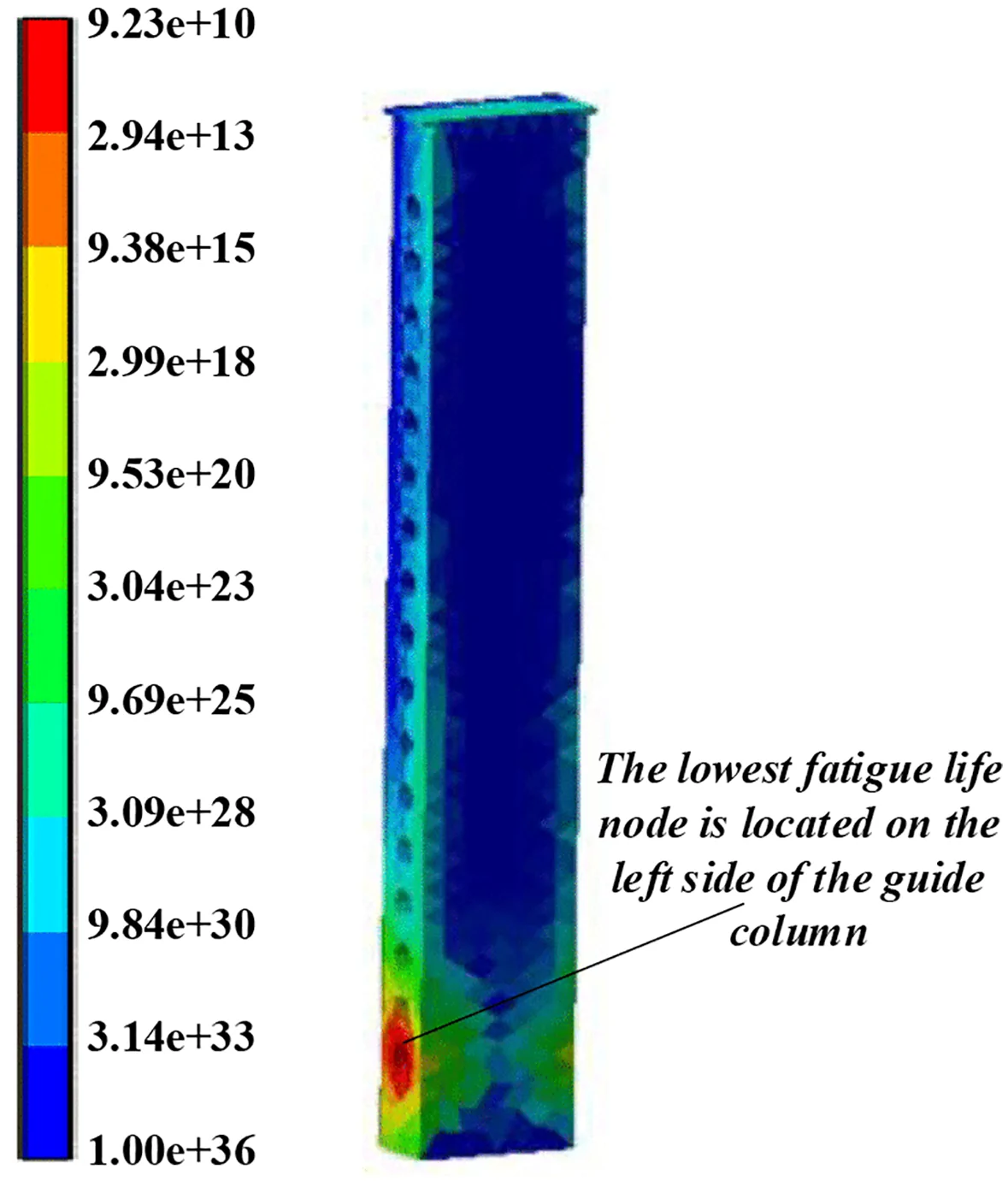
Fatigue-life contour of the guide column during the forward stroke.

The Durability module embedded in RecurDyn was used to analyze the fatigue life of the guide column during the forward stroke based on the stress results obtained from the rigid–flexible coupled dynamic simulation. According to the rainflow counting method and the Palmgren–Miner criterion, the fatigue-life contour of the guide column under the forward-stroke condition was obtained, as shown in Fig 8. The fatigue-critical region of the guide column was mainly located at the contact positions between the limiting holes on both sides and the upper surface of the hydraulic rod. The minimum life was 9.23 × 10^10^ cycles.

According to cumulative damage theory, the damage of the guide column during the forward stroke was accumulated as follows:


$$\begin{array}{c} D=\frac{\text{1}}{N_{q}} \end{array}$$ (1)

The corresponding fatigue life is expressed as:


$$\begin{array}{c} N=\frac{\text{1}}{D} \end{array}$$ (2)

where *D* is the damage value, *N*_*q*_ is the fatigue life under the forward-stroke condition, and *N* is the fatigue life corresponding to the minimum number of cycles.

The calculation results show that the damage value was 1.12 × 10^−11^, corresponding to a fatigue life of 8.93 × 10^10^ cycles. This result satisfies the fatigue-life design requirement of the height-adjustment mechanism.

## 4. Topology optimization of the height-adjustment mechanism

Because the shearer cable bending test machine needs to undergo more than 9,000 cyclic dragging motions in a single test, the mass of the height-adjustment mechanism affects not only the energy consumption of the whole machine but also the fatigue life of key components. The dynamic simulation results show that the stresses of all key components of the height-adjustment mechanism were significantly lower than the allowable stress of the material, indicating a certain degree of material redundancy. Therefore, on the premise of ensuring reliability, the guide column, limit block, supporting plate, slider and guide-column sleeve were selected as optimization objects, and topology optimization was adopted for lightweight design.

### 4.1 Methods used in numerical studies

This paper uses the variable density method for topological optimization, also known as the SIMP method. The core idea is to set element density as a continuous design variable to build a more precise and flexible optimization model, with values ranging from 0 to 1.

The functions constructed by the SIMP interpolation model are shown in equation (3):


$$\begin{array}{c} E=\rho^{p}E_{\text{0}} \end{array}$$ (3)

Where: *E* is the elastic modulus of the material unit being punished, MPa; *E*_0_ is the actual elastic modulus of the material element, MPa; ρ is the density of the unit; *P* is the penalty factor.

The variable density optimization model, with flexibility minimization (stiffness maximization) as the objective function and volume constraints as constraints, is:


$$\begin{array}{c} \left\{ \begin{array}{c} @l@minC=U^{T}KU=\sum_{e=1}^{N}{\left(\rho_{e}\right)}^{p}u_{e}^{T}k_{d}\mu_{e}\\ subject\;to\left\{ \begin{array}{c} @l@V=f_{p}V_{\text{0}}=\sum_{e=1}^{N}\rho_{e}\nu_{e}\leq V\\ KU=F\\ 0\leq \rho_{min}\leq \rho_{e}\leq \rho_{max} \end{array}\right. \end{array}\right. \end{array}$$ (4)

Where: *C* is the structural deformation energy; ρe is the relative density of the e-th unit; *K* is the total stiffness matrix; *k*_d_ is the stiffness matrix of the d-th element; *F* is the force vector; *U* is the displacement vector; $\mu_{e}$ is the displacement vector of the e-th cell; *N* is the total number of units; *v*_e_ is the volume of the e-th cell; *V* is the volume of the material filled; *V*_0_ is the volume of the design domain; *f*_p_ is the optimized volume factor.

When using this algorithm, its iterative convergence rate is significantly better than conventional methods, and computational accuracy can maintain a 0.1% error threshold even in complex geometric topology optimization. Especially when handling large-scale topology optimization problems, it ensures both computational accuracy and computational stability.

The objective function of the Lagrange function, as shown in equation (5):


$$\begin{array}{c} L=C+\lambda_{\text{0}}\left(V-f_{\nu }V_{\text{0}}\right)+\lambda_{\text{1}}^{T}\left(KU-F\right)+\sum_{e=1}^{N}\lambda_{\text{2}}^{c}\left(\rho_{min}-\rho_{e}\right)+\sum_{e=1}^{N}\lambda_{\text{3}}^{c}\left(\rho_{e}-\rho_{max}\right) \end{array}$$ (5)

Where: *λ*_0_, *λ*_1_, *λ*_2_ and *λ*_3_ are all Lagrange multipliers.

In constraint optimization problems, the $\rho_{e}$ necessary criterion for the objective function to reach an extremum is given by the Kuhn-Tucker condition, and its mathematical expression must satisfy the following form:


$$\begin{array}{c} \left\{ \begin{array}{c} @l@\frac{\partial C}{\partial \rho_{e}}+\lambda_{\text{0}}\frac{\partial V}{\partial \rho_{e}}+\lambda_{\text{1}}^{T}\frac{\partial \left(KU\right)}{\partial \rho_{e}}=0\;\;\rho_{min}\leq \rho_{e}\leq \rho_{max}\\ \frac{\partial C}{\partial \rho_{e}}+\lambda_{\text{0}}\frac{\partial V}{\partial \rho_{e}}+\lambda_{\text{1}}^{T}\frac{\partial \left(KU\right)}{\partial \rho_{e}}\geq 0\;\;\rho_{e}=\rho_{min}\\ \frac{\partial C}{\partial \rho_{e}}+\lambda_{\text{0}}\frac{\partial V}{\partial \rho_{e}}+\lambda_{\text{1}}^{T}\frac{\partial \left(KU\right)}{\partial \rho_{e}}\leq 0\\ V=f_{V}\cdot V_{\text{0}}\;\;\rho_{e}=\rho_{max}\\ F=KU\\ \lambda_{\text{2}}^{e}>0,\lambda_{\text{3}}^{e}>0,e=1,2,\ldots N \end{array}\right. \end{array}$$ (6)

Therefore, if $\rho_{e}=\rho_{max}$, the upper limit constraint fails, then $\lambda_{\text{2}}^{e}=\text{0}$, $\lambda_{\text{3}}^{e}\geq \text{0}$; if $\rho_{min}<\rho_{e}<\rho_{max}$ the upper and lower limits constraints of the design variable fail, then $\lambda_{\text{2}}^{e}=\lambda_{\text{3}}^{e}=\text{0}$; if $\rho_{e}=\rho_{min}$, the lower limit constraint fails, then $\lambda_{\text{2}}^{e}>\text{0}$, $\lambda_{\text{3}}^{e}=\text{0}$.

In summary, Kuhn-Tucker equals:


$$\begin{array}{c} \left\{ \begin{array}{c} @l@\frac{\partial L}{\partial \rho_{e}}=\frac{\partial C}{\partial \rho_{e}}+\lambda_{\text{0}}\frac{\partial V}{\partial \rho_{e}}+\lambda_{\text{1}}^{T}\frac{\partial \left(KU\right)}{\partial \rho_{e}}\lambda_{\text{2}}^{e}+\lambda_{\text{3}}^{e}=0\\ V=f_{V}\cdot V_{\text{0}}\\ F=KU\\ \lambda_{\text{2}}^{e}\left(\rho_{min}-\rho_{e}\right)=0\\ \lambda_{\text{3}}^{e}\left(\rho_{e}-\rho_{max}\right)=0\\ \lambda_{\text{2}}^{e}>0,\lambda_{\text{3}}^{e}>0,e=1,2,\ldots N\\ \rho_{min}\leq \rho_{e}\leq \rho_{max} \end{array}\right. \end{array}$$ (7)

Consider $\frac{\partial C}{\partial \rho_{e}}+\lambda_{\text{0}}\frac{\partial V}{\partial \rho_{e}}+\lambda_{\text{1}}^{T}\frac{\partial \left(KU\right)}{\partial \rho_{e}}=0$ And consider the situation $C=U^{T}KU$ Available:


$$\begin{array}{c} \frac{\partial U^{T}}{\partial \rho_{e}}KU+U^{T}\frac{\partial K}{\partial \rho_{e}}U+U^{T}K\frac{\partial U}{\partial \rho_{e}}+\lambda_{\text{0}}\frac{\partial V}{\partial \rho_{e}}+\lambda_{\text{1}}^{T}\left(\frac{\partial K}{\partial \rho_{e}}U+K\frac{\partial U}{\partial \rho_{e}}\right)=0 \end{array}$$ (8)

Organized:


$$\begin{array}{c} \frac{\partial U^{T}}{\partial \rho_{e}}\left(2KU+K\lambda_{\text{1}}\right)+\lambda_{\text{1}}^{T}\frac{\partial K}{\partial \rho_{e}}U+p{\left(\rho_{e}\right)}^{P-1}u_{e}^{T}k_{\text{0}}u_{e}+\lambda_{\text{0}}\nu_{e}=0 \end{array}$$ (9)

Since λ_1_ is a column vector, take the value λ_1_ = −2U, and substitute into the above formula to get:


$$\begin{array}{c} -p{\left(\rho_{e}\right)}^{p-1}u_{e}^{T}k_{\text{0}}u_{e}+\lambda_{\text{0}}\nu_{e}=0 \end{array}$$ (10)

From the above formula, it can be obtained:


$$\begin{array}{c} \frac{p{\left(\rho_{e}\right)}^{p-1}u_{e}^{T}k_{\text{0}}u_{e}}{\lambda_{\text{0}}\nu_{e}}=1 \end{array}$$ (11)

When system flexibility reaches an extreme value, a unique energy distribution pattern appears within the design domain: at this time, the strain energy density field exhibits uniform distribution, specifically as the global energy density tends to equalize. Based on this conclusion, a form of iterative updates for design variables can be further established [26]:


$$\begin{array}{c} \rho_{N+1}=\rho_{\epsilon }^{N}B^{\eta } \end{array}$$ (12)

$$\begin{array}{c} B=\frac{p{\left(\rho_{e}\right)}^{p-1}u_{e}^{T}k_{\text{0}}u_{e}}{\lambda_{\text{0}}\nu_{e}} \end{array}$$ (13)

$$\begin{array}{c} \rho_{N+1}=\left\{ \begin{array}{c} @l@max\left(\rho_{min},\rho_{e}^{N}-m\right)\;\;if\rho_{e}^{N}B^{\eta }<max\left(\rho_{min},\rho_{e}^{N}-m\right)\\ \rho_{\text{e}}^{\text{N}}{\text{B}}^{\eta }\;\;ifmax\left(\rho_{min},\rho_{e}^{N}-m\right)<\rho_{e}^{N}B^{\eta }<min\left(1,\rho_{e}^{N}+m\right)\\ min\left(1,\rho_{e}^{N}+m\right)\;\;if\rho_{e}^{N}B^{\eta }>min\left(1,\rho_{e}^{N}+m\right) \end{array}\right.\; \end{array}$$ (14)

Where: *m* is the movement limit; η is the numerical damping coefficient.

### 4.2 Topology optimization model and parameter settings

**Fig 9:**
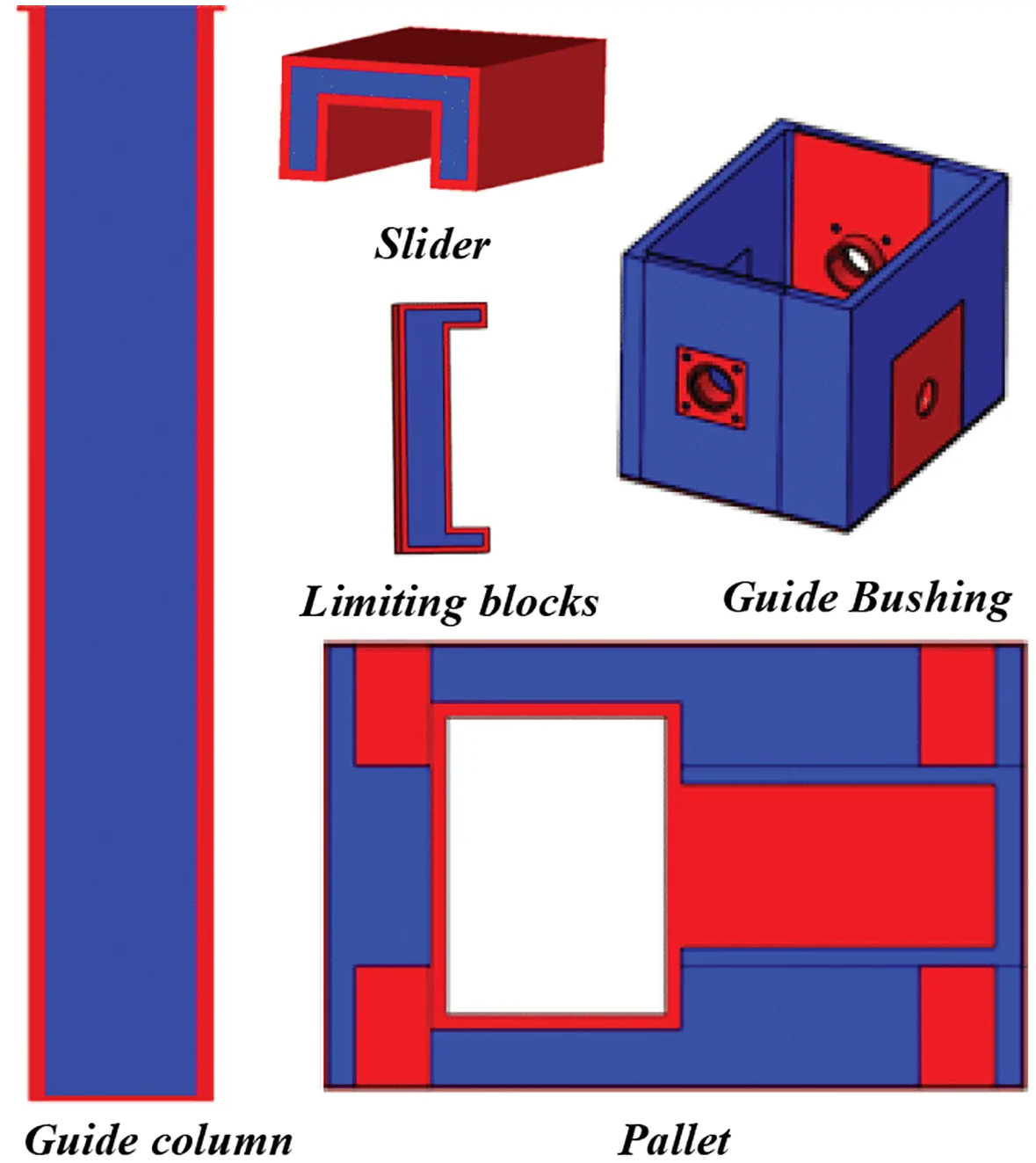
Division of design and non-design domains for each component of the height-adjustment mechanism.

A topology optimization model of the key components of the height-adjustment mechanism was established based on OptiStruct. Before optimization, geometric cleanup and mesh generation were performed for each component. The contact surfaces, bolt holes and assembly functional surfaces were defined as non-design domains, while the internal material-redundant regions were defined as design domains. Taking the guide column as an example, as shown in Fig 9, the left and right sides of the guide column are in contact with the limit blocks, the lower surface is in contact with the cable connector, and the surfaces of the limiting holes are in contact with the hydraulic rod. Therefore, these positions were defined as non-design regions, while the internal region of the guide column was defined as the design region. In Fig 9, red represents the non-design region and blue represents the design region.

In OptiStruct, the topology optimization area uses the PSOLID solid element type, i.e., a three-dimensional solid finite element element. Since the structure of each component of the height adjustment mechanism is symmetrical and the load distribution is basically symmetrical, symmetry constraints are introduced during the optimization process to improve optimization efficiency and ensure the structural rationality of the optimization results. The guide post, limiting block, and slider are set with double-sided symmetry constraints, while the support plate and guide post sleeve are set with single-sided symmetry constraints.

**Fig 10:**
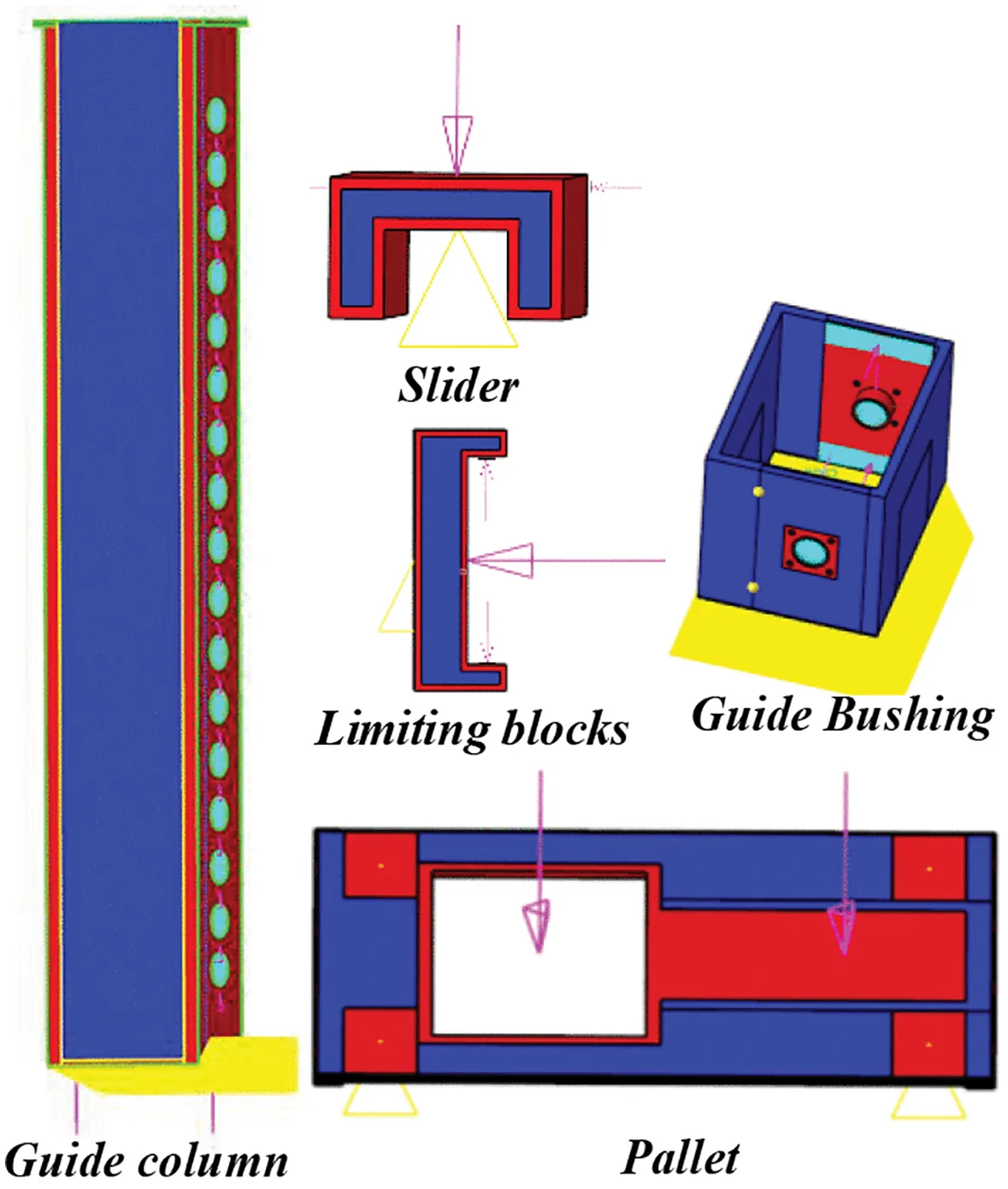
Loads and constraints applied to each component of the height-adjustment mechanism.

To ensure that the optimized structure satisfies the actual loading requirements, the loads and boundary constraints applied to each component were kept consistent with the maximum loading state of the corresponding component in the dynamic analysis. The bottom end of the guide column was fixed, and equivalent loads were applied to the remaining components according to their actual assembly and force-transmission relationships, as shown in Fig 10.

**Fig 11:**
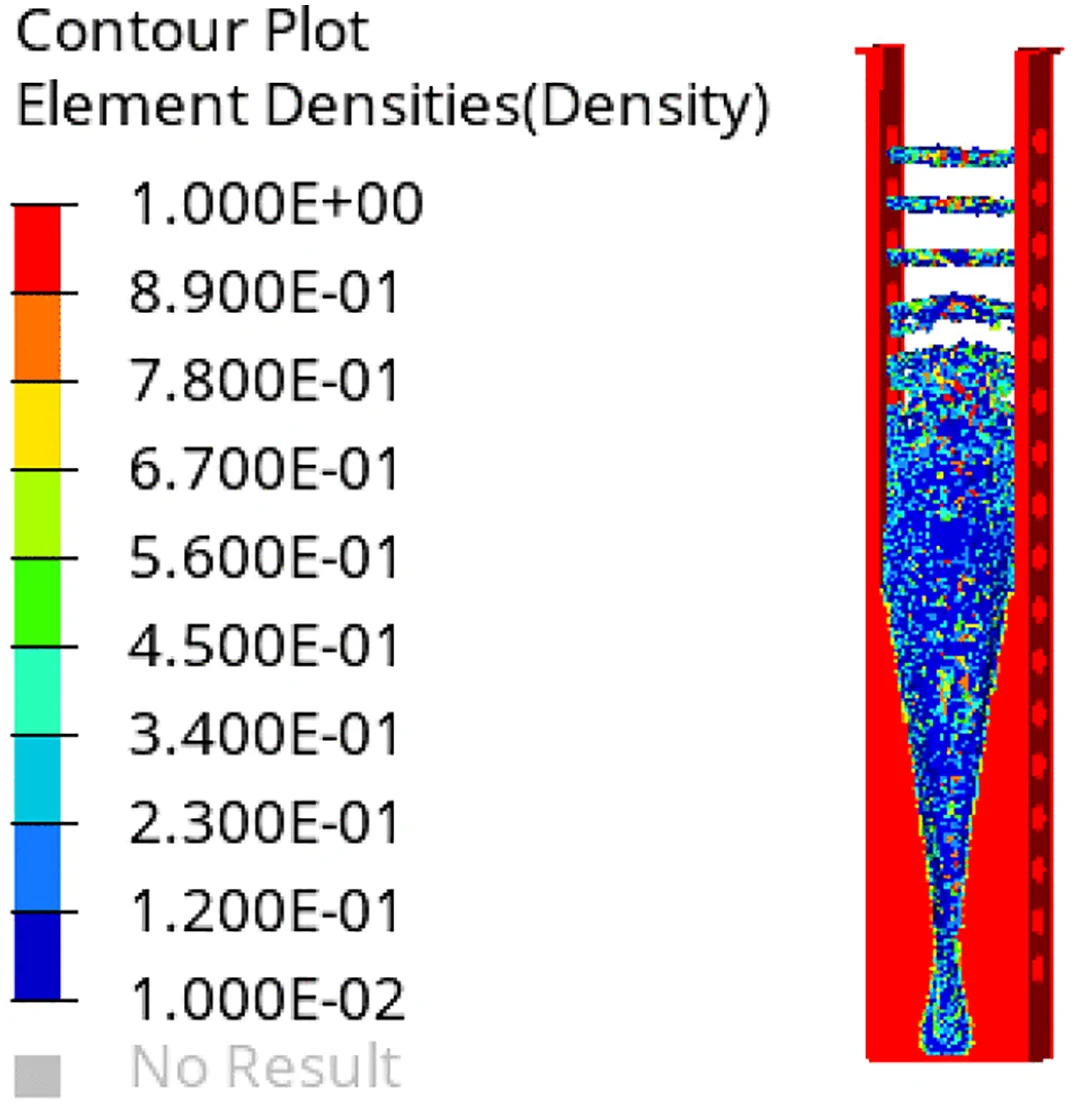
Compliance-based density contour of the topology-optimized guide column.

**Fig 12:**
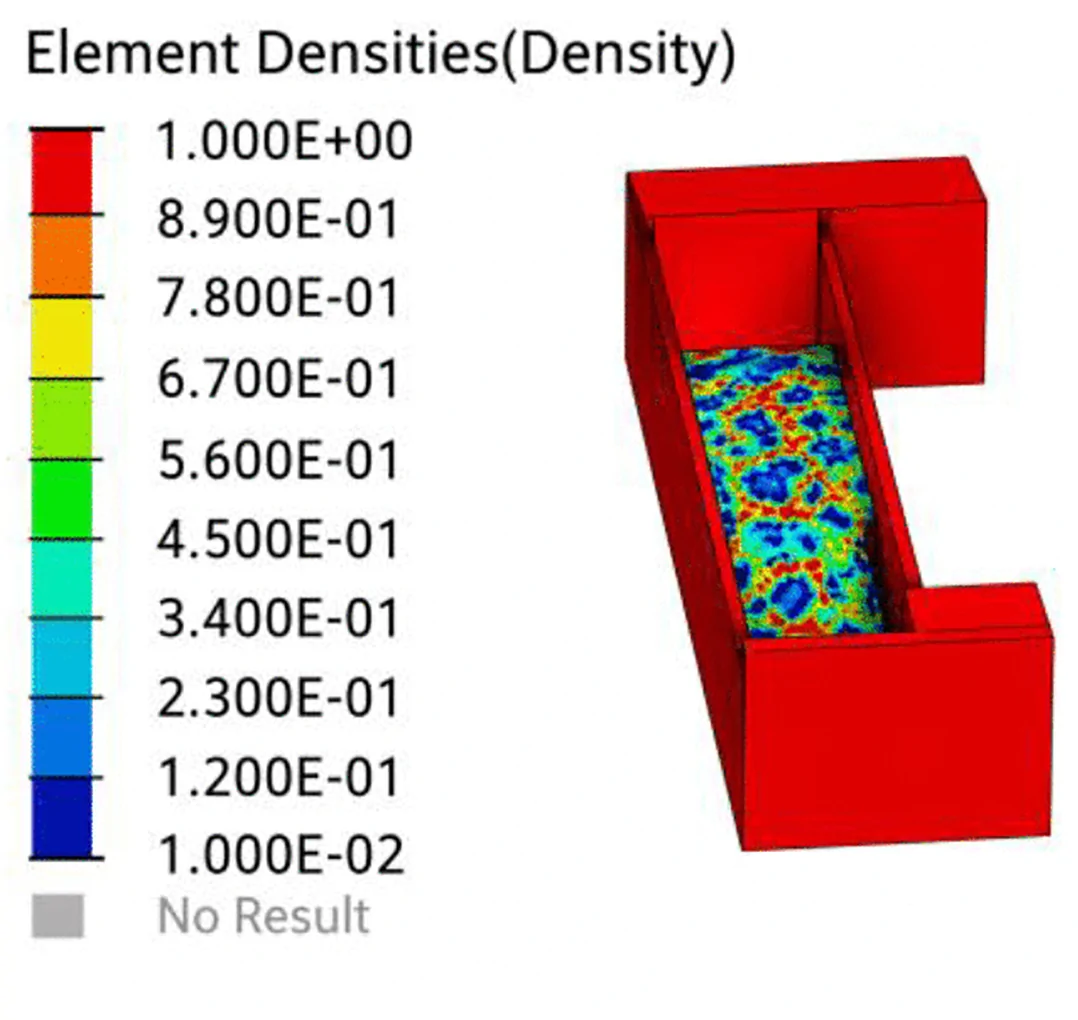
Compliance-based density contour of the topology-optimized limit block.

**Fig 13:**
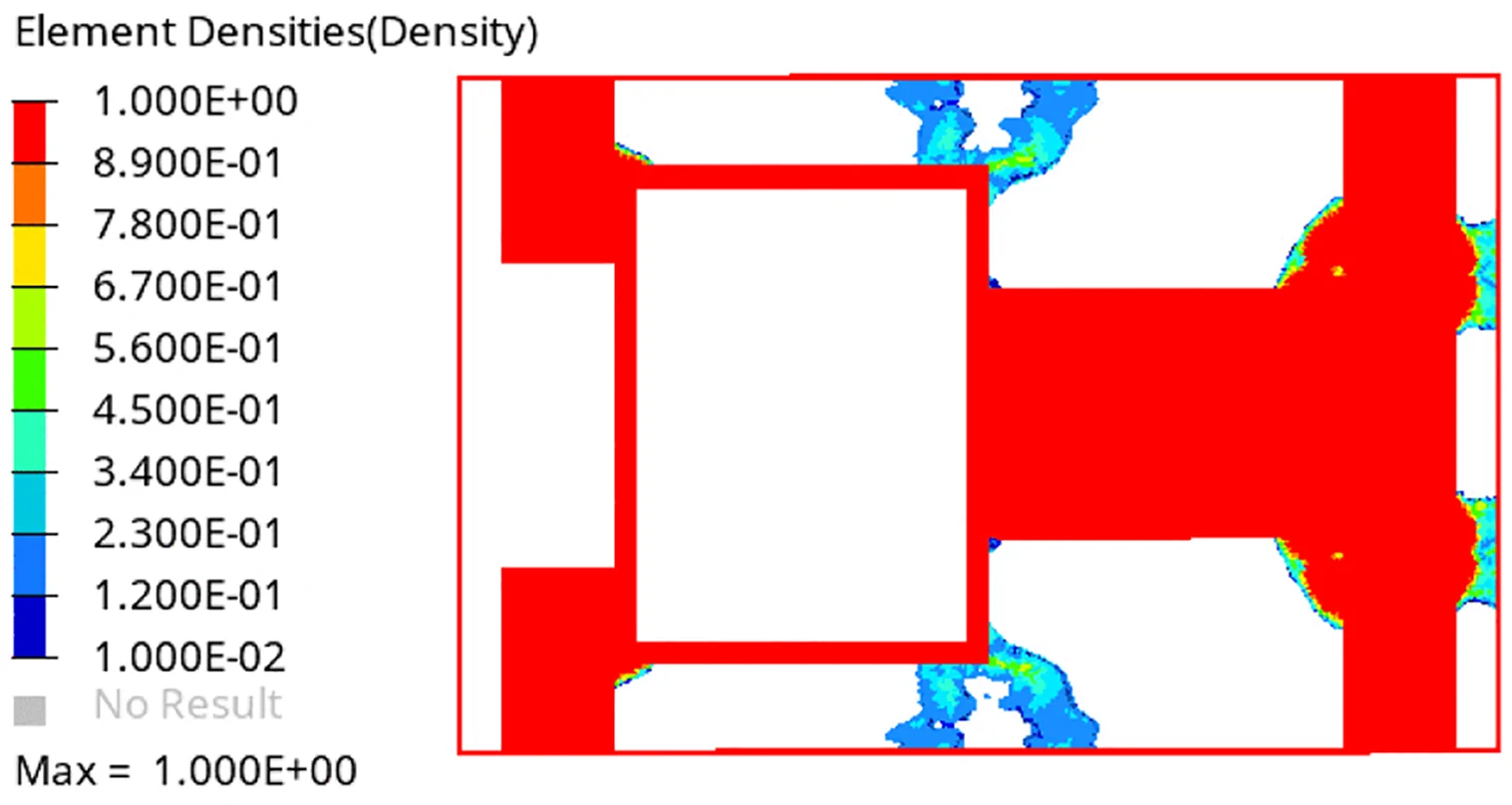
Compliance-based density contour of the topology-optimized supporting plate.

**Fig 14:**
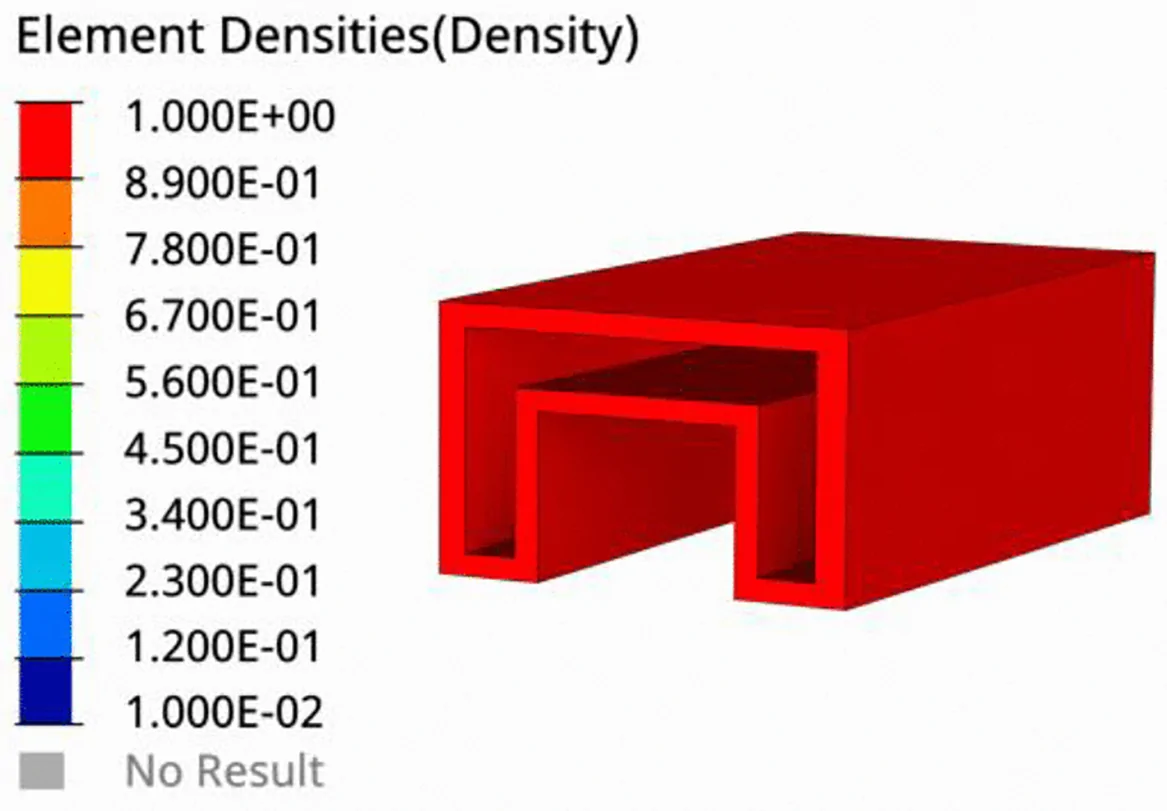
Compliance-based density contour of the topology-optimized slider.

**Fig 15:**
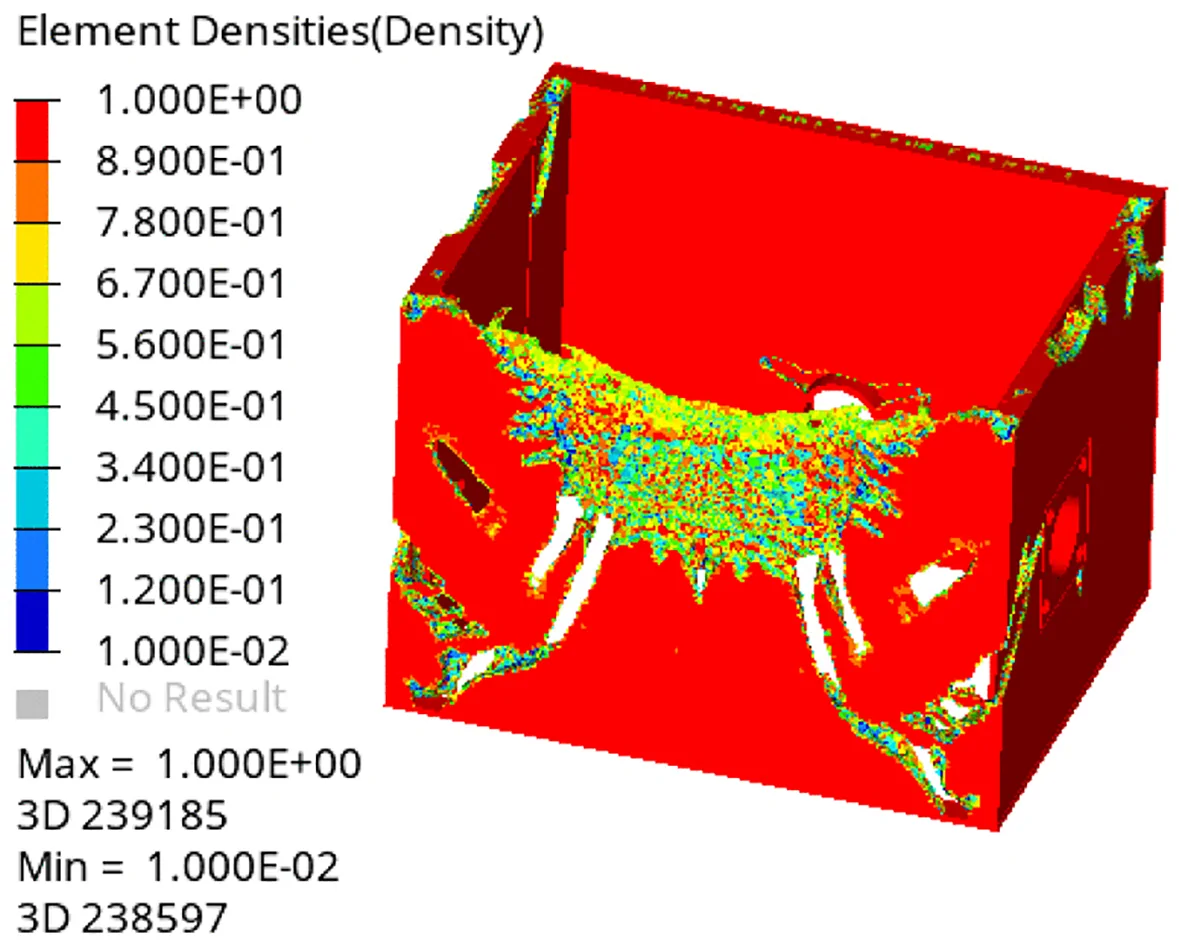
Compliance-based density contour of the topology-optimized guide-column sleeve.

In the optimization parameter settings, minimum compliance was taken as the objective, and volume fraction was taken as the constraint. Stress constraints, symmetry constraints, draw-direction constraints and checkerboard control were also introduced as manufacturing constraints. The topology optimization module in OptiStruct was used to solve the optimization problems of the components [27]. The density contours of the topology-optimized components are shown in Figs 11–15.

The topology optimization results show that material was mainly retained along the key load-transfer paths and in the main load-bearing regions, while material in non-critical regions was effectively removed. Specifically, the material of the guide column was mainly retained in the bottom load-bearing region. Most of the non-load-bearing material inside the slider was removed. In contrast, because the guide-column sleeve had many loaded positions, most of the material in its design domain was retained.

### 4.3 Dynamic analysis after optimization

**Fig 16:**
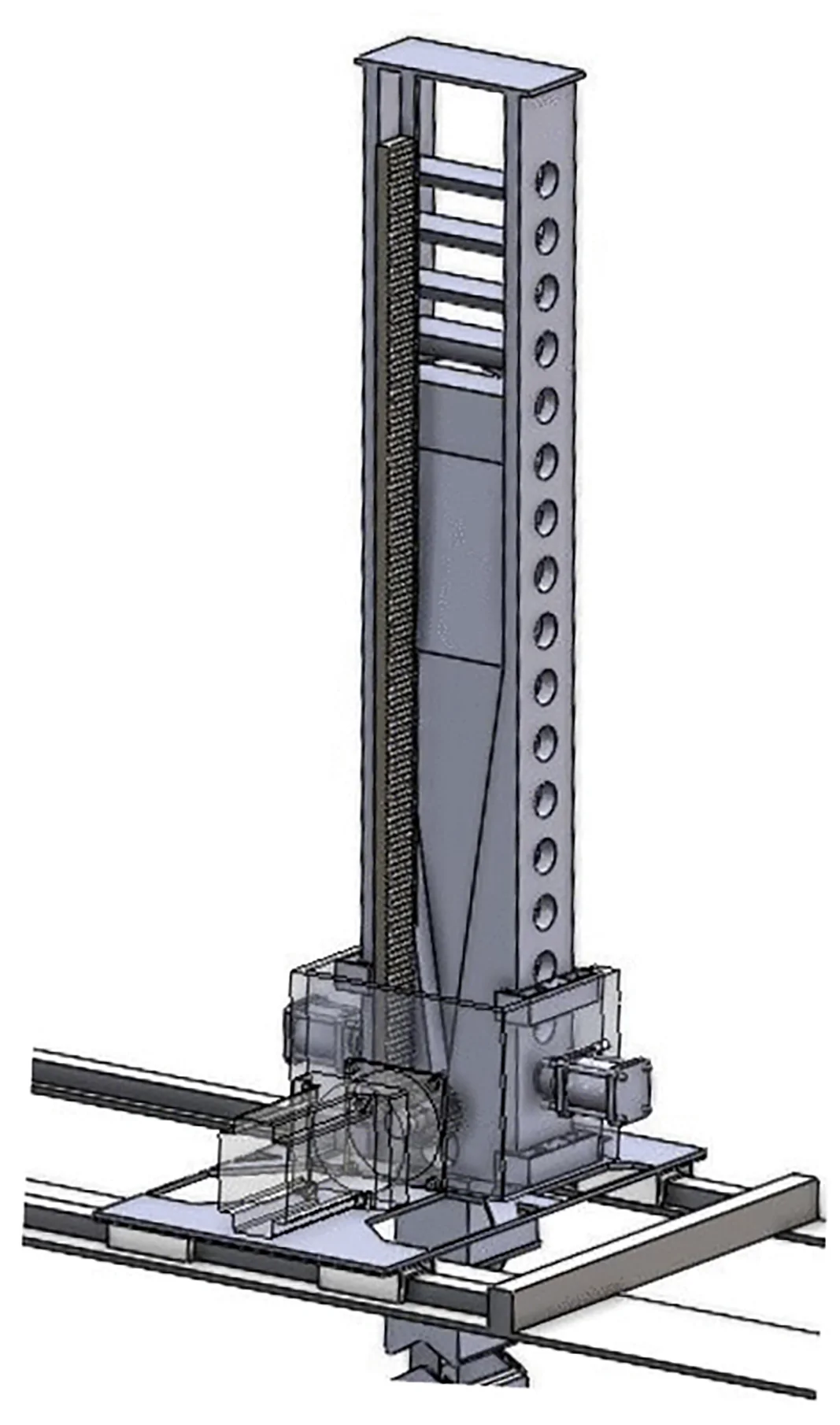
Assembly of the topology-optimized height-adjustment mechanism.

To further verify the service performance of the topology-optimized structure under actual operating conditions, the reconstructed components were substituted into the original rigid–flexible coupled model of the height-adjustment mechanism. Dynamic simulations were then conducted in RecurDyn under the same operating conditions as those before optimization. The optimized assembly of the height-adjustment mechanism is shown in Fig 16. The SolidWorks software was used to assign material properties to the models before and after optimization, and the quality attributes of the five component models were evaluated. The comparison of mass values for each mechanism component before and after optimization is presented in Table 1.

**Table 1:** Comparison of maximum equivalent stress of key components before and after optimization.

Name	Quality before optimization (kg)	Optimized quality (kg)	Poor quality (kg)	Change rate (%)
guiding pillar	255.6	159.2	96.4	−37.7
Limit block	3.6	1.6	2	−55.6
Tray	36.3	23.1	13.2	−36.4
Slider	5.2	2.5	2.7	−51.9
Guide post sleeve	74.8	70.6	4.2	−5.6

**Fig 17:**
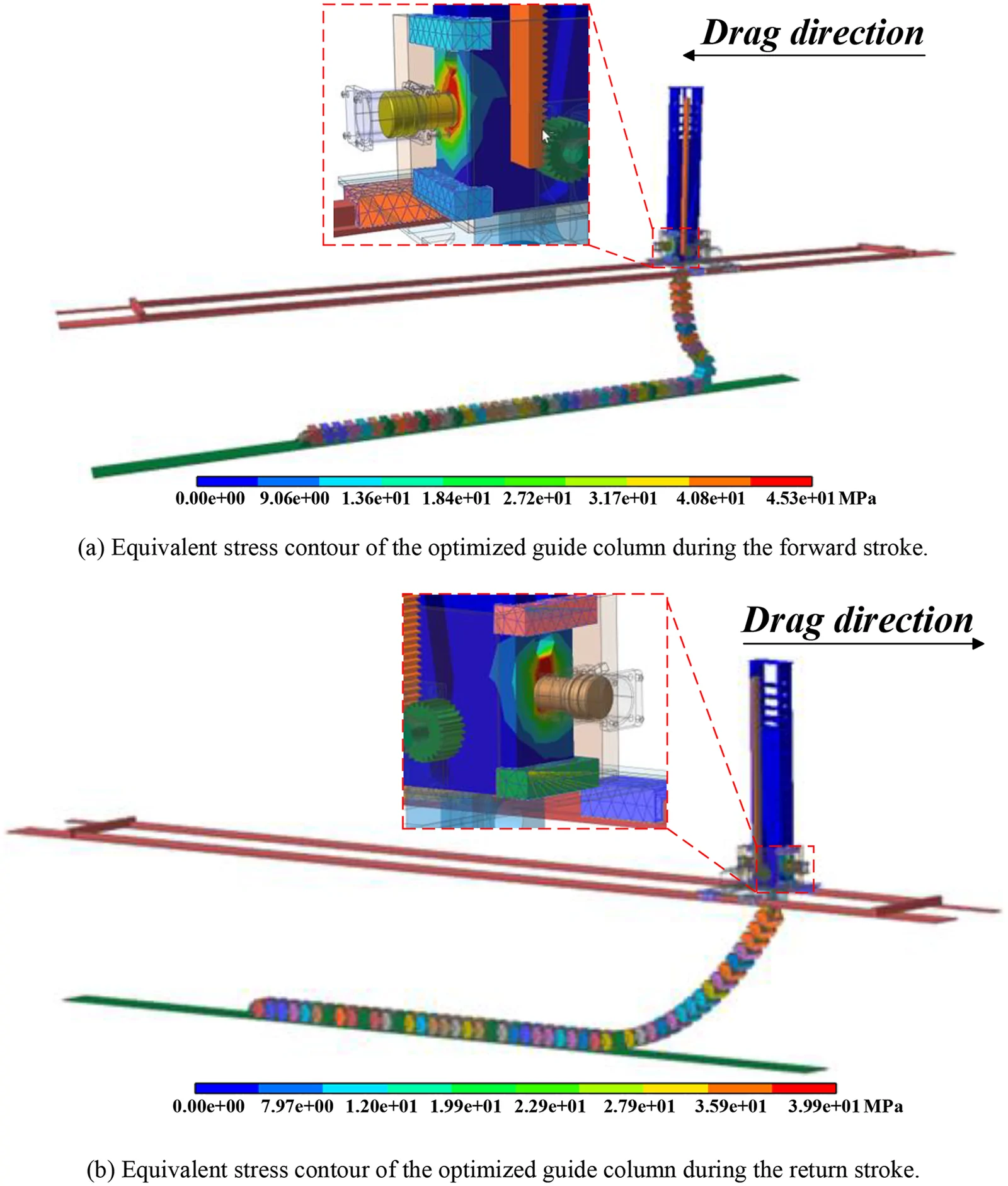
Equivalent stress contours of the optimized guide column. (a) Equivalent stress contour of the optimized guide column during the forward stroke. (b) Equivalent stress contour of the optimized guide column during the return stroke.

**Fig 18:**
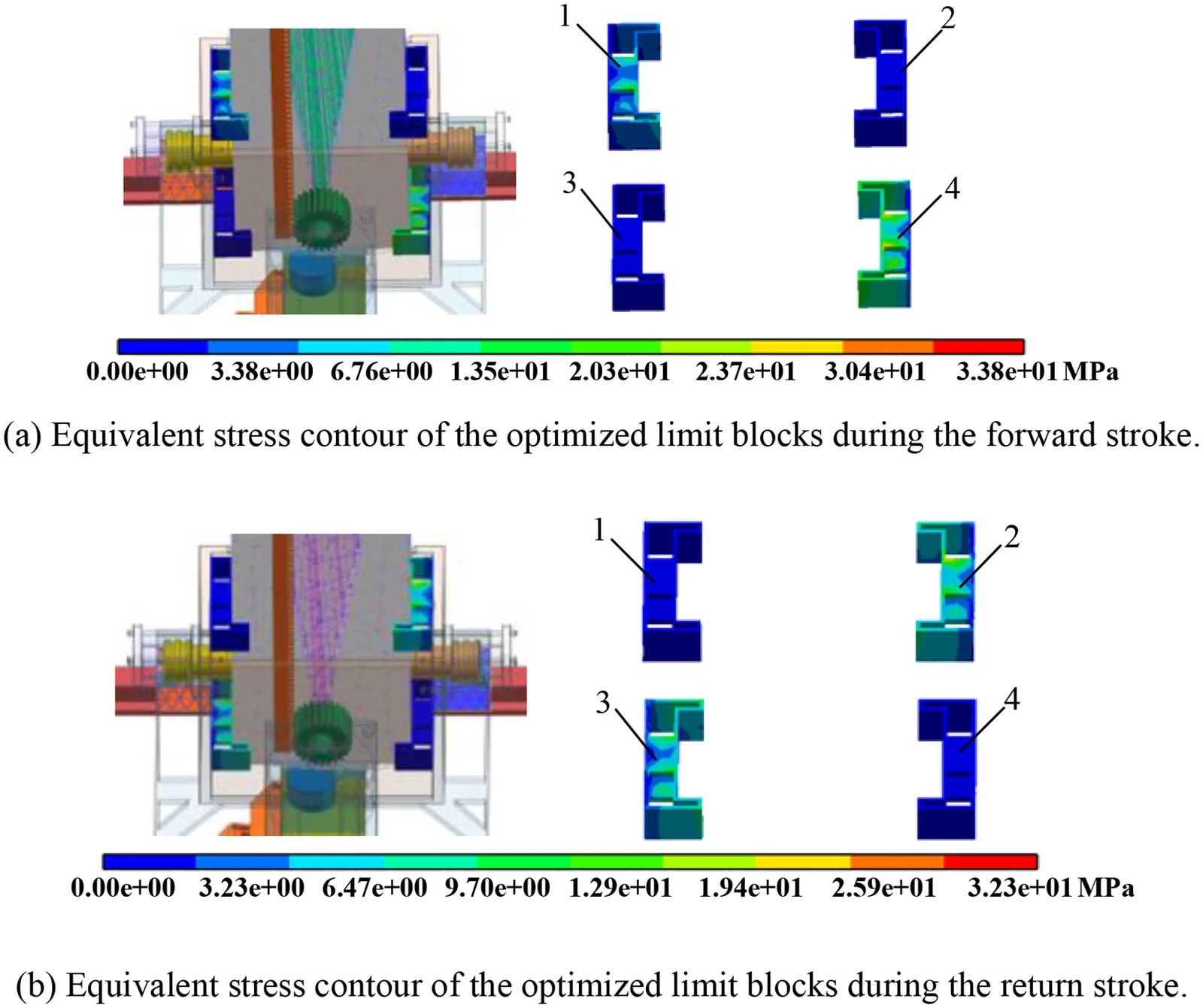
Equivalent stress contours of the optimized limit blocks. (a) Equivalent stress contour of the optimized limit blocks during the forward stroke. (b) Equivalent stress contour of the optimized limit blocks during the return stroke.

**Fig 19:**
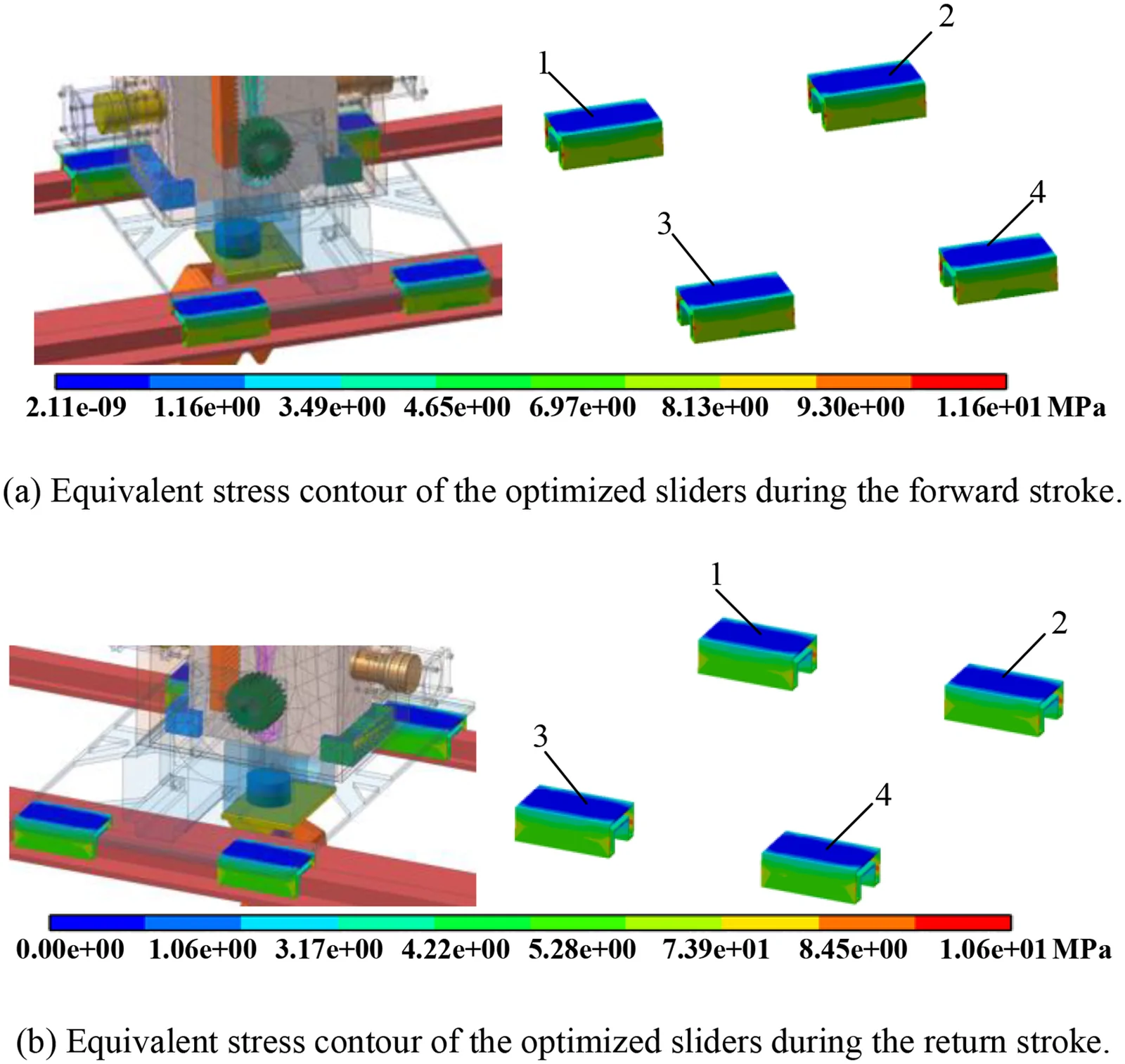
Equivalent stress contours of the optimized sliders. (a) Equivalent stress contour of the optimized sliders during the forward stroke. (b) Equivalent stress contour of the optimized sliders during the return stroke.

The equivalent stress contours of the optimized guide column, limit blocks and sliders are shown in Figs 17–19. The results show that the high-stress region of the guide column was still mainly located near the limiting holes on both sides of its bottom end. The maximum equivalent stresses under the forward and return stroke conditions were 49.96 MPa and 39.87 MPa, respectively. The maximum equivalent stresses of the limit blocks were 33.79 MPa and 32.33 MPa, respectively. The maximum equivalent stresses of the sliders were 11.62 MPa and 10.56 MPa, respectively.

The comparison of the maximum equivalent stresses of each component before and after optimization is shown in Table 2. Compared with the original structure, the maximum equivalent stresses of the guide column, limit block and slider under the same operating conditions were reduced by 23.3%, 26.4% and 34.5%, respectively. This indicates that the topology-optimized structure not only achieved mass reduction but also improved the dynamic loading state of key components during actual operation.

**Table 2:** Comparison of maximum equivalent stress of key components before and after optimization.

Component	The maximum equivalent stress of the forward stroke/MPa		Maximum equivalent stress on the return stroke/MPa	
	Before optimization	After optimization	Before optimization	After optimization
Guide column	65.11	49.96	63.24	39.87
Limiting blocks	45.89	33.79	43.77	32.33
slider	17.73	11.62	15.04	10.56

### 4.4 Fatigue-life analysis after optimization

**Fig 20:**
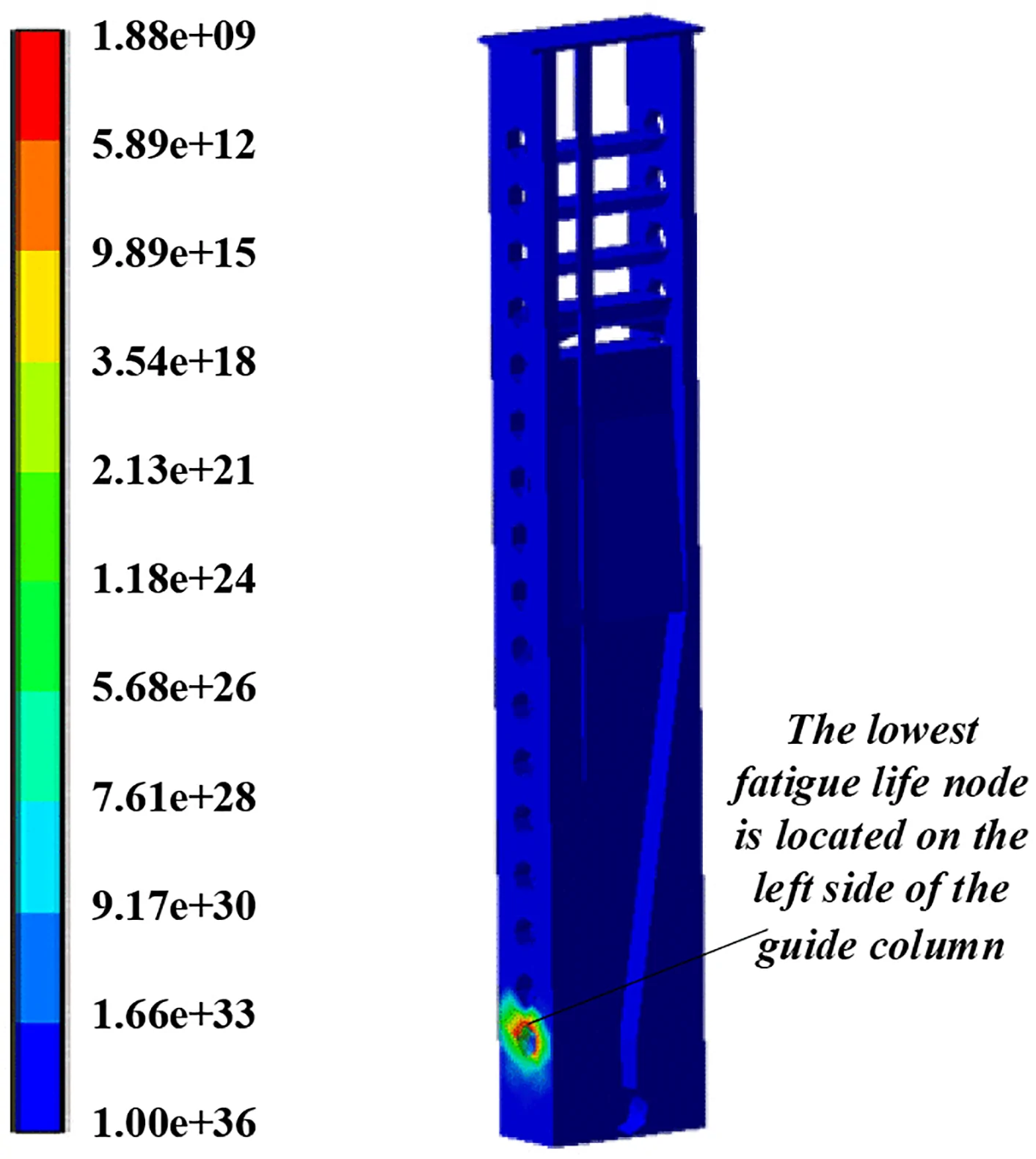
Fatigue-life contour of the optimized guide column during the forward stroke.

The dynamic results show that the optimized guide column was still the component most significantly subjected to alternating loads in the height-adjustment mechanism. Therefore, the guide column was continuously selected for fatigue-life prediction. The Durability module was used to perform fatigue analysis of the optimized guide column, and the fatigue-life contour is shown in Fig 20. The results show that the fatigue-critical region was still located near the limiting holes on the left and right sides of the bottom end of the guide column. The minimum number of cycles during the forward stroke was 1.88 × 10⁹. After cumulative damage calculation for the forward stroke, the damage value was 6.31 × 10^−10^, corresponding to a fatigue life of 1.58 × 10⁹ cycles.

Compared with the pre-optimization fatigue life of 8.93 × 10^10^ cycles, the fatigue life of the optimized guide column decreased to some extent, but it still satisfied the fatigue-life design requirement of the height-adjustment mechanism. This indicates that topology optimization significantly reduced structural mass while satisfying the fatigue performance of key components under cyclic loading.

## 5. Conclusions

(1) To ensure consistency between the bending-resistance test of shearer cables and actual underground operating conditions, this study addressed the limitation that existing test machines can only conduct fixed-height dragging tests. A hydraulically assisted height-adjustment system based on rack-and-pinion transmission was designed. Through the coordinated operation of the height-adjustment system and the dragging system, the test machine can match the dragging heights required by different specifications of shearer cables.
(2) The height-adjustment state and the cable dragging–bending state were selected as two dynamic simulation conditions. When the mechanism was raised to 1.8 m, the maximum equivalent stresses of the gear and rack were 73.27 MPa and 42.82 MPa, respectively. During the cable dragging–bending test, the maximum equivalent stresses of the guide column, limit block and slider were 65.11 MPa, 45.89 MPa and 17.73 MPa, respectively. The dynamic simulation results show that the guide column was subjected to the highest maximum equivalent stress during the forward stroke, and alternating cyclic loads acted between the guide column and the hydraulic rod during operation, whereas the equivalent-stress curves of the other components were smooth. Fatigue-life prediction was carried out for the guide column during the forward stroke. Based on cumulative damage theory, the fatigue life was calculated as 8.93 × 10^10^ cycles, satisfying the long-term service requirement of the height-adjustment mechanism.
(3) The variable-density topology optimization method based on OptiStruct reduced the total mass of the height-adjustment mechanism by 132.6 kg while satisfying the strength and stiffness requirements. The maximum dynamic stresses of the guide column, limit block and slider were reduced by 23.3%, 26.4% and 34.5%, respectively. The fatigue life of the guide column was 1.58 × 10⁹ cycles, satisfying the fatigue-life design requirement of the height-adjustment mechanism. Therefore, a balance between structural performance and lightweight design was achieved.
(4) From the perspective of industrial application, the topology optimization of the height-adjustment mechanism has clear potential benefits in terms of weight reduction, cost saving and structural performance. The total mass of the optimized mechanism was reduced by 132.6 kg, which can decrease material consumption, machining workload, transportation difficulty and installation effort. For manufacturers and users of shearer cable bending test machines, the lighter mechanism can also reduce the driving load of the height-adjustment system, lower energy consumption during repeated dragging tests and decrease wear of transmission and supporting components. In addition, because the optimized material distribution was mainly retained along the key load-transfer paths, the load-bearing efficiency and rigidity utilization of the assembly mechanism were improved. The maximum equivalent stresses of the guide column, limit block and slider were reduced by 23.3%, 26.4% and 34.5%, respectively, and the optimized guide column still satisfied the fatigue-life design requirement. Therefore, the proposed topology optimization method provides practical value for developing lighter, more economical and more reliable shearer cable bending test machines.
